# Spermidine: a physiological autophagy inducer acting as an anti-aging vitamin in humans?

**DOI:** 10.1080/15548627.2018.1530929

**Published:** 2018-10-11

**Authors:** Frank Madeo, Maria A. Bauer, Didac Carmona-Gutierrez, Guido Kroemer

**Affiliations:** a Institute of Molecular Biosciences, University of Graz, Graz, Austria; b BioTechMed Graz, Graz, Austria; c Equipe 11 labellisée Ligue contre le Cancer, Centre de Recherche des Cordeliers, INSERM U, Paris, France; d Metabolomics and Cell Biology Platforms, Gustave Roussy Comprehensive Cancer Center, Villejuif, France; e Sorbonne Paris Cité, Université Paris Descartes, Paris, France; f Université Pierre et Marie Curie, Paris, France; g Pôle de Biologie, Hôpital Européen Georges Pompidou, Paris, France; h Department of Women’s and Children’s Health, Karolinska University Hospital, Stockholm, Sweden

**Keywords:** Autophagy, cancer, cardiovascular diseases, health span extension, longevity

## Abstract

Spermidine is a natural polyamine that stimulates cytoprotective macroautophagy/autophagy. External supplementation of spermidine extends lifespan and health span across species, including in yeast, nematodes, flies and mice. In humans, spermidine levels decline with aging, and a possible connection between reduced endogenous spermidine concentrations and age-related deterioration has been suggested. Recent epidemiological data support this notion, showing that an increased uptake of this polyamine with spermidine-rich food diminishes overall mortality associated with cardiovascular diseases and cancer. Here, we discuss nutritional and other possible routes to counteract the age-mediated decline of spermidine levels.

Spermidine is a natural polyamine present in all living organisms that is critically involved in the maintenance of cellular homeostasis. This chemical affects numerous biological processes, including cell growth and proliferation, tissue regeneration, DNA and RNA stabilization, enzymatic modulation, and regulation of translation, among others [1–3]. Furthermore, spermidine exhibits anti-inflammatory and antioxidant properties, enhances mitochondrial metabolic function and respiration, promotes chaperone activity and improves proteostasis [4]. Intriguingly, external supplementation of spermidine exerts various beneficial effects on aging and age-related disease in a variety of model organisms, including mice [5,6]. For example, spermidine feeding extends lifespan across species [5,7,8], promotes cardio- [5] and neuroprotection [9–11], stimulates antineoplastic immune response [12] and may avoid immunosenescence by stimulating memory T-cell formation [13,14]. Many of these anti-aging properties have been causally linked to the capacity of spermidine to ensure proteostasis through the stimulation of cytoprotective macroautophagy [15–17]. Age-associated conditions including cancer, neurodegeneration and cardiovascular diseases are directly connected to the intracellular accumulation of toxic debris, and its removal by autophagy constitutes a well-documented avenue for protection against age and disease [18–20].

Spermidine induces autophagy through the inhibition of several acetyltransferases [7], including EP300 [21], one of the main negative regulators of autophagy [22]. Its potency has been recently quantified to be equivalent to that of rapamycin [23], an FDA-approved immunosuppressant with protective and autophagy-stimulatory properties [24,25]. Importantly, the genetic impairment of autophagy abrogates the beneficial effects of spermidine on longevity of yeast, flies and worms [7]. Moreover, in mice, the cardioprotective and immunosurveillance-associated anticancer effects of spermidine are lost when autophagy is inhibited in myocardial or cancer cells, respectively [5,12].

In general, polyamine levels in individual organisms are highly diverse. Nevertheless, one commonality is that tissue concentrations of spermidine decline in an age-dependent manner in both model organisms and humans [7,9,26,27]. This may account for decreased autophagy and drive the onset of age-associated diseases. A remarkable exception to this decline in spermidine levels are healthy nonagenarians and centenarians, who retain whole-blood concentrations reminiscent of younger (middle-aged) individuals [26]. The optimal concentration of spermidine in humans to maintain optimal autophagy levels for healthy aging, however, still needs further investigation. The age-induced spermidine decline must involve the alteration of one or several of the distinct factors that determine systemic availability of spermidine (Figure 1). In principle, spermidine bioavailability is determined by the 3 different sources of this polyamine: (i) cellular biosynthesis, (ii) production by intestinal microorganisms and (iii) nutritional supply, as well as (iv) catabolism and (v) urinary excretion.

**Figure 1.**
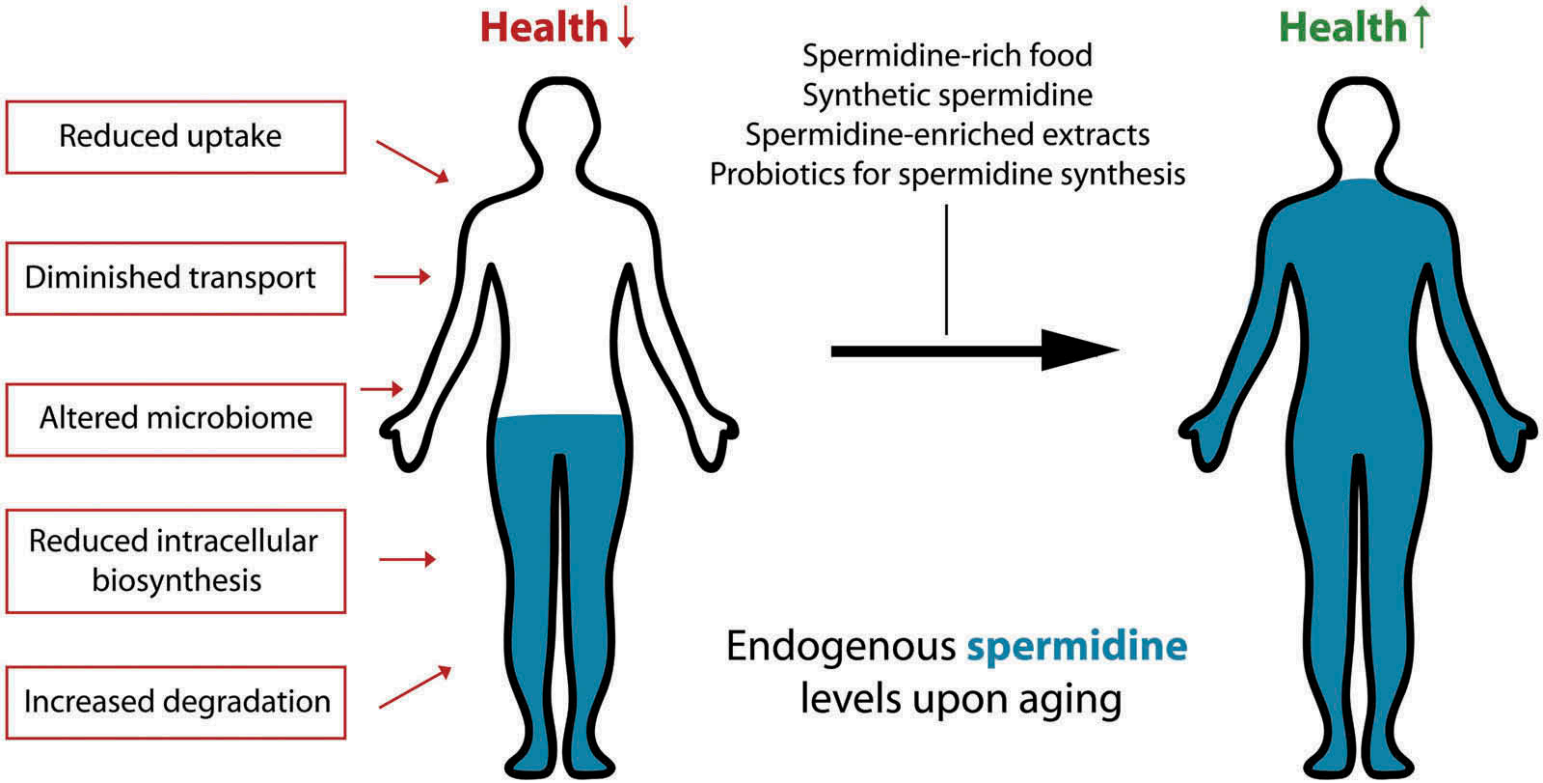
Possible routes to counteract the age-induced decline of spermidine levels. With old age, endogenous spermidine concentrations diminish due to alterations in one or several factors that determine the bioavailability of the substance in the body. This detrimental decline may be counteracted by ingesting polyamine-rich food items, polyamine-enriched plant extracts, synthetic spermidine, or by stimulating polyamine synthesis in the gut microbiome through supplementation of prebiotics or probiotics.

Within mammalian cells, spermidine is generated from its precursor putrescine (which itself is generated from ornithine) or by oxidative degradation of spermine [2,3,28]. The net cytosolic spermidine content further depends on its catabolism, which is mainly driven by acetylation and subsequent oxidation [29–31], but also on its uptake from the extracellular space and its excretion by the cell. This may be mediated by membrane transporters such as the ones operating in yeast and bacteria [32], but could also involve endocytosis/exocytosis processes. Still, polyamine transport in mammals remains not well understood and needs further examination.

The intestinal microbiota represents another source of spermidine synthesis within our body. In mice, the concentration of spermidine in the gut lumen has been shown to directly depend on the colonic microbiota [33]. Thus, commensal gut bacteria may regulate polyamine concentration in the human intestine [34]. In fact, preclinical studies support the critical involvement of the gut microbiome in generating health-relevant spermidine. Therefore, blood levels of spermidine and spermine can be upregulated through oral administration of the polyamine-producing probiotic *Bifidobacterium* LKM512, resulting in suppressed inflammation and improved longevity in old mice [35]. Interestingly, these effects can be enhanced, if combined with additional supplementation of arginine, a polyamine precursor [36]. Thus, arginine may be considered as a prebiotic for stimulating intestinal spermidine synthesis. However, other prebiotic strategies may consist of providing agents that favor the selective expansion of polyamine-producing bacteria or the upregulation of spermidine synthesis by the existing gut microbiota.

Dietary spermidine is rapidly resorbed from the intestine and distributed in the body without degradation [37]. Thus, food items with high spermidine content can contribute to raising the bioavailability of this polyamine. Such spermidine-rich food items comprise unprocessed plant-derived foods including the durian fruit, shitake mushrooms, fresh green pepper, wheat germ, amaranth grain, cauliflower and broccoli, just to mention a few, but also products resulting from fermentation processes that involve polyamine-generating bacteria and fungi, e.g. soybean products such as natto or many types of mature cheese [38]. Therefore, the systemic levels of spermidine are influenced by diet either directly, by the ingestion of polyamine-rich food items, or indirectly, by effects of the diet on the spermidine-producing microbiota.

The use of a spermidine-rich diet to elevate systemic spermidine levels in human organs [39] may constitute a promising strategy for promoting healthy aging. Spermidine shows no adverse effects during life-long administration in mice [5], and a currently ongoing clinical trial on supplementation of spermidine-rich plant extracts to elderly persons indicates good safety and tolerability [40]. Recently, for the first time, an epidemiological study has reported a positive association between nutritional spermidine uptake and human health span and lifespan [38]. A total of 829 participants aged 45–84 were observed over a time frame of 15 years, during which 341 became deceased. The spermidine uptake of each participant was calculated based on food-frequency questionnaires every 5 years. Individuals with high total nutritional spermidine uptake displayed a reduced incidence of cancer and cardiovascular diseases, correlating with an overall improved survival. This was even the case after correction for possibly confounding factors such as age, sex, body mass index, alcohol and aspirin consumption, dietary quality, metabolic diseases, physical activity, and socioeconomic status.

Importantly, the association between high dietary spermidine uptake and reduced mortality has been recapitulated in a second, independent cohort of 1770 healthy participants aged 39–67 with a medium follow-up of 13 years [38]. However, one limitation of the aforementioned epidemiological studies resides in the absence of laboratory assessments of spermidine concentrations. It will be important to perform similar observational studies while correlating the plasma spermidine levels of each individual patient with dietary patterns (and other lifestyle factors), ideally in a longitudinal fashion. It would be particularly interesting to correlate these measurements with the quantification of autophagic flux and protein acetylation levels, which can be determined in peripheral blood mononuclear cells [41], hoping to establish an epidemiological triangulation between spermidine, autophagy and health.

The age-protective effects of increased spermidine intake are in line with the paradigm that a decline of spermidine concentration upon aging is not only causally linked to reduced health- and lifespan, but that it might be reversed. Given the factors that determine spermidine levels in the body (Figure 1), what would be the most feasible avenue to reverse this age-dependent decline? Despite its documented low toxicity in mice and humans [5,39], an ‘overdose’ of spermidine might, at least theoretically, compromise cellular homeostasis if supraphysiological concentrations are reached. Instead, it may be a more feasible strategy to restore the levels of spermidine that are declining in the elderly to physiological levels reminiscent of young(er) individuals. This may be achieved by a polyamine-rich diet, as demonstrated by Kiechl et al. [38], with food extracts rich in spermidine, supplementation of synthetic spermidine, or prebiotics and probiotics that drive microbial polyamine synthesis in the intestine.

In sum, in our view, spermidine is synthesized by our organism in sufficient quantities during youth, but not in old age. Thus, one may argue that, as we age, spermidine evolves to the status of a vitamin, and thus has to be supplemented from external sources to secure the maintenance of autophagic flux required for organismal homeostasis.
